# Prevalence of acute watery diarrhea in vulnerable children, mothers’ knowledge, and management: a cross-sectional study from Yemen

**DOI:** 10.3389/fped.2026.1859345

**Published:** 2026-06-10

**Authors:** Mansour Abdu Al-Taj

**Affiliations:** Department of Community Medicine, Faculty of Medicine and Health Sciences, Sana’a University, Sana’a, Yemen

**Keywords:** children, diarrhea, health-seeking behaviors, knowledge, mother, preventions, risk factors, Yemen

## Abstract

**Background:**

Diarrhea remains one of the leading causes of morbidity and mortality among children worldwide. Enabling mothers with knowledge and resources is essential for mitigating the negative impacts of diarrhea in children in resource-limited contexts.

**Objective:**

The aim of this study is to measure the prevalence of acute watery diarrhea in children under five years old in vulnerable communities. It also aims to assess mothers’ knowledge of the route of transmission and prevention of diarrhea in children and to identify the methods used to manage diarrhea.

**Methods:**

This study employed a cross-sectional design using a structured questionnaire. A total of 895 mothers from internally displaced and host communities were interviewed face-to-face. Data were analyzed using Stata version 16, with descriptive statistics presented as proportions. Bivariate analysis was conducted using the chi-square test, and multivariable logistic regression was used to estimate the adjusted odds ratios (AOR) and compute 95% confidence intervals (CI).

**Results:**

The proportion of children with acute watery diarrhea was 36.4%. Only 39.3% of mothers had good knowledge about the route of transmission and prevention of diarrhea in children. 15.9% of them provided their children with oral rehydration solution (ORS) during the last episode of diarrhea. Mothers aged 40 years or above (AOR = 1.80; 95% C1 1.139–2.86), and those who had secondary school or higher education (AOR = 1.95; 95% C1 1.35–2.82) were more likely to have good knowledge about childhood diarrhea. Mothers who had good knowledge were nearly twice as likely to give ORS to children with acute watery diarrhea (AOR = 1.69; 95% CI: 1.15–2.49).

**Conclusion:**

Acute watery diarrhea in young, vulnerable children remains high. The findings of this study highlight a concerning gap in mothers’ knowledge regarding the route of transmission and prevention of diarrhea in children, as well as a low rate of ORS use. These findings emphasize the importance of targeted educational interventions to improve mothers’ knowledge of diarrhea prevention in children and to enhance home management of childhood diarrhea in vulnerable communities.

## Introduction

Diarrheal disease is the third leading cause of death among children aged 1–59 months. The number of deaths from diarrhea in children under five years old dropped by 83%, from an estimated 2.7 million in 1980 to about 443,833 in 2021 (1–3). The majority occurred in low- and middle-income countries (4, 5). The decline in fatalities can be attributed in part to better treatments, including oral rehydration therapy and zinc supplementation (6). Moreover, diarrhea is responsible for a high burden of malnutrition and stunted growth in children (7, 8). The majority of diarrhea-related fatalities are attributed to the loss of fluids and electrolytes. Factors such as poor sanitation (9, 10), contaminated water sources (9, 11), lack of handwashing with water and soap during the critical times (9), and limited hygiene services (12) found to be associated with the childhood diarrhea.

Preventive measures are vital for minimizing the incidence of diarrhea in children, while proper management of childhood diarrhea at home particularly in remote area is critical for reducing mortality rates linked to this condition. Approximately 60% of all diarrheal fatalities can have been prevented by improving access to clean drinking water, sanitation services, and hygiene (13). Several intervention studies have found that increasing the knowledge of mothers and caregivers about risk factors and preventive measures such as treating drinking water, safely disposing of child feces, and practicing proper sanitation and handwashing at critical times significantly improves hygiene practices (14–16) and reduces the occurrence of childhood diarrhea (16, 17). Understanding the knowledge of mothers or caregivers and the factors influencing their awareness of diarrhea risk factors and prevention is crucial for effective diarrhea prevention and management, as they often serve as the primary decision-makers in managing childhood diarrhea. The majority of studies assessing caregivers’ knowledge and practices regarding diarrhea in children have been conducted within the general population and often involve small sample sizes (18–20). This gap underlines the need for further research with larger sample sizes, specifically focusing on vulnerable populations.

Yemen is a country where childhood illness are prevalent, particularly among vulnerable populations (21–23). Recent study indicates that the prevalence of diarrhea among under five years of internally displaced children in Yemen was 40.4% (22). In remote, neglected locations such as IDP camps, access to health services is extremely limited, making prevention and home management of childhood illnesses like diarrhea the only means of protecting children’s health. This study aimed to measure the prevalence of acute watery diarrhea among children under five years of age and assess mothers’ knowledge about diarrhea in both internally displaced and host communities. It identified factors that influenced mothers’ knowledge and examined their health-seeking behaviors when a child developed diarrhea.

## Methods

### Study design, and setting, and population

A cross-sectional survey was conducted in February 2024. The survey was conducted in five districts across four Yemeni governorates characterized by substantial concentrations of internally displaced persons (IDPs). The districts included Al-Qahirah and Al-Mudhaffar in Taiz, Broum in Hadramaut, Al-Wadi in Marib, and Khab Al-Sha’af in Al-Jawf. The study population involved families from internally displaced and host communities in the selected districts. Households were eligible if they had at least one child aged 6–59 months who lived in the same household with their mother.

### Sample size calculation and sampling technique

The sample size for this study was calculated using the single-proportion formula. This study is part of a broader survey assessing WASH-KAP, as well as child health indicators. To determine the most appropriate proportion for the calculation, the prevalence of acute malnutrition was selected as the best proxy for the estimated proportion in the formula. Based on the Yemeni National Health and Demographic Survey (24), the proportion of acute malnutrition among children under five was estimated at 16%, with a 95% confidence level and a significance level of 0.05. The initial sample size obtained was 206. To account for the clustering effect, the sample size was multiplied by a design effect of 2, yielding a revised estimate of 412 households. Since the study included two distinct populations (IDPs and host communities) the sample size was further doubled, resulting in an adjusted figure of 824 households. Allowing for an anticipated non-response rate of 5%, the sample size increased to 867 households, and was subsequently further increased to 895 households. The final sample size was allocated proportionally between IDPs and host communities, ensuring adequate representation of both groups. Accordingly, 627 households were allocated to IDPs and 268 to host communities.

Two stage sampling was conducted to select the participants. In the first stage, local authorities prepared lists of enumeration areas for both IDPs and host communities. Thereafter, 21 clusters for IDPs and 9 clusters for host communities were selected from these lists using systematic random sampling, with an estimated average of approximately 30 households per cluster. In the second stage, approximately 30 households per cluster were selected using the World Health Organization’s expanded immunization method (25).

### Data collection

Face-to-face interviews were conducted with mothers using a structured questionnaire by trained female data collectors. Mothers were asked about demographic factors, their knowledge of route of transmission and prevention of diarrhea in their children, as well as their health-seeking behaviors when their children experienced diarrhea most recently. The questionnaire used in this study was adapted from the Yemen Demographic Health Survey (24), and United Nations High Commissioner for Refugees WASH KAP survey questionnaires (26, 27). It was translated from English into Arabic and piloted on 60 women from IDPs and host communities. The pilot study was conducted to assess the clarity and cultural appropriateness of the questionnaire items, and no major issues were identified. The translated questionnaire was reviewed and validated by the ethics committee of the Medical College and Health Sciences at Sana’a University. Data collectors were recruited from the same areas as the study participants and administered the questionnaire using local accents to enhance rapport and improve the quality of data collection.

### Variables

Our primary outcomes were acute watery diarrhea in children under five years of age, mothers’ knowledge of diarrhea and the management of childhood diarrhea. Acute watery diarrhea was defined as the passage of three or more loose, watery stools within a 24-hour period during the two weeks preceding the interview (28). Overall knowledge was evaluated using 10 items on route of transmission and prevention of diarrhea and was categorized as either poor or good. The knowledge items on routes transmission of diarrhea in children included: drinking contaminated water; eating contaminated food; flies; eating with unclean hands; open-field defecation; and bottle feeding. The knowledge items on prevention included: treating drinking water; washing hands after using the latrine; washing hands before feeding the child; and using a latrine for defecation. Mothers responded “yes” or “no” to each item.

To assess the management behaviors for a child with diarrhea, mothers were asked how they handled the situation the last time their child experienced diarrhea. Respondents mentioned various methods, including giving ORS, yogurt, rice water, and water as well as seeking health services. Other responses included giving herbal remedies, and consulting traditional healers.

Independent factors included the age of the mother, categorized as ≤ 25 years, 26–30 years, 31–39 years, and ≥ 40 years. Mothers’ education levels were classified as no education, primary, and secondary and above, and mothers’ employment status was noted as either “yes” or “no.” The number of children born to mothers was categorized as ≤ 2, 3–4, and ≥ 5. Housing types were classified as either Independent/Flat or Hut/Tent. Respondents were asked to report whether the household owned a television and a smart-phone.

### Data analysis

Stata version 16 was used for data cleaning and analysis. Percentages were used to describe the categorical variables. The ten knowledge items were reduced using principal component analysis with varimax rotation. A predictive score was then generated using the median score of the first component as the cutoff. Scores above the median were classified as indicating good knowledge, whereas scores below the median indicated poor knowledge. Principal component analysis was used to generate weighted scores based on each item’s contribution to the overall construct. Internal consistency was assessed using Cronbach’s alpha, which was 0.7, demonstrating good reliability. A bivariate analysis using chi-square test was conducted to identify factors associated with mothers’ good knowledge of diarrhea. All variables with a *p*-value below 0.05 were included in the multivariable analysis using logistic regression.

## Result

Almost a quarter of mothers were aged 25 years or younger, and 22.9% had secondary school or higher education. Additionally, 92.0% were housewives, and around one-third had five or more children. More than half of the families lived in tents or huts, and 62.7% owned television (Table 1).

**Table 1:** Characteristics of respondents and households.

Characteristics	number	percent
Mother's age (Years)		
≤ 25	226	25.2
26–30	260	29.1
31–39	250	27.9
≥ 40	159	17.8
Mother's education		
No education	418	46.7
Primary	272	30.4
Secondary and above	205	22.9
Mother's work		
No	823	92.0
Yes	72	8.0
Number of children born to mother		
≤ 2	263	29.4
3–4	361	40.3
≥ 5	271	30.3
Type of house		
Independent/Flat	431	48.2
Hut/Tent	464	51.8
Family owned television		
Yes	561	62.7
No	334	37.3
Family owned smart-phone		
Yes	828	92.5
No	67	7.5
Type of family		
Internally displaced	627	70.1
Host community	268	29.9

The prevalence of acute watery diarrhea among children under five years was 36.4%. A higher prevalence of acute diarrhea was observed among children residing in Taiz governorate (47.9%), followed by children residing in Marib governorate (40.3%). The overall rate of mothers demonstrating good knowledge about diarrhea was 39.3%. Among the knowledge on causes or transmission routes of diarrhea in children, defecating in the open field (15.3%) and using bottle-feeding (7.8%) were less commonly recognized by mothers. In contrast, eating dirty food was well known (64.0%). Around 33.0% of mothers were aware that handwashing after using the latrine, and before feeding the child can protect against diarrhea. Only 16.9% reported that using latrine for defecation can prevent diarrhea in children (Table 2).

**Table 2:** Prevalence of acute watery diarrhea in children and mothers’ knowledge and management of childhood diarrhea.

Items	Number	Percentage
Child had acute diarrhea		
Yes	326	36.4
No	569	63.6
Distribution of acute diarrhea by current place of residence		
Taiz	115	47.9
Hadramout	24	16.2
Marib	144	40.3
Al-Jwaf	43	28.7
Diarrhea Knowledge index		
Poor knowledge	543	60.7
Good knowledge	352	39.3
mode of transmission of diarrhea		
Drinking contaminated water	310	34.6
Eating dirty food	573	64.0
Flies	541	60.4
Eating with dirty hands	278	31.1
Defecating in the open field	137	15.3
bottle-feeding	70	7.8
Method for prevention of diarrhea		
Treating drinking water	287	32.1
Washing hands after latrine	302	33.7
Washing hands before feeding the child	301	33.6
Using latrine for defecation	151	16.9
Management of diarrhea		
Providing oral rehydration solution	142	15.9
Providing yogurt	464	51.8
Providing rice water	40	4.5
Providing water	19	2.1
Visiting health facilities	187	20.9
Others	43	4.8

When mothers were asked what they did the last time their children had diarrhea, the majority (51.8%) reported that they provided yogurt. Only 15.9% mentioned giving ORS, and 20.9% indicated that they sought medical care (Table 2).

In the bivariate analysis, mother’s age (*p* < 0.001), mother’s education (*p* < 0.001), mother’s work (*p* < 0.001), type of house (*p* < 0.001), and family owned television (*p* = 0.014) were significantly associated with mothers’ knowledge about diarrhea (Table 3). Additionally mothers from the host community had significantly better knowledge compared with mothers from displaced communities (*p* < 0.001).

**Table 3:** Distribution of respondents and household characteristics by mothers’ level of knowledge.

Characteristics	Good knowledge		Poor knowledge		*p*. value
	*n*	%	*n*	%	
Mother's age (Years)					
≤ 25	77	34.1	149	65.9	<0.001
26–30	81	31.1	179	68.9	
31–39	114	45.6	136	54.4	
≥ 40	80	50.3	79	49.7	
Mother's education					
No education	153	36.6	265	63.4	<0.001
Primary	93	34.2	179	65.8	
Secondary and above	106	51.7	99	48.3	
Mother's work					
No	309	37.6	514	62.4	<0.001
Yes	43	59.7	29	40.3	
Number of children born to mother					
≤ 2	98	37.3	165	62.7	0.639
3–4	148	41.0	213	59.0	
≥ 5	106	39.1	165	60.9	
Type of house					
Independent/Flat	200	46.40	231	53.60	<0.001
Tent/Hut	152	32.76	312	67.24	
Family owned television					
Yes	238	42.4	323	57.6	0.014
No	114	34.1	220	65.9	
Family owned smart-phone					
Yes	321	38.8	507	61.2	0.227
No	31	46.3	36	53.7	
Type of family					
Host community	135	50.4	133	49.6	<0.001
Internally displaced	217	34.6	410	65.4	

n; number, %; percentage.

After adjusting for covariates, mothers aged 40 years or older had nearly double the odds of having good knowledge (AOR = 1.80; 95% C1 1.139–2.86) compared with mothers aged 25 years or younger; similarly, mothers with secondary education or higher had approximately double the odds of good knowledge (AOR = 1.95; 95% C1 1.35–2.82) compared with mothers with no formal education. The associations between mothers’ knowledge and mothers’ work, type of house, household television ownership, and family type disappeared (Table 4).

**Table 4:** Factors influencing mothers’ knowledge of diarrhea transmission and prevention in children.

Characteristics	COR (95% CI)	*P*. value	AOR (95% CI)	*P*. value
Mother's age (Years)				
≤ 25	Ref.		Ref.	
26–30	0.87 (0.60–1.28)	0.494	0.82 (0.55–1.22)	0.334
31–39	1.62 (1.12–2.35)	0.011	1.48 (0.99–2.20)	0.053
≥ 40	1.96 (1.29–2.97)	0.001	1.80 (1.139–2.86)	0.012
Mother's education				
No education	Ref.		Ref.	
Primary	0.90 (0.65–1.24)	0.518	0.98 (0.69–1.38)	0.901
Secondary and above	1.85 (1.32–2.60)	<0.001	1.95 (1.35–2.82)	<0.001
Mother's work				
No	Ref.		Ref.	
Yes	2.47 (1.51–4.03)	<0.001	1.59 (0.94–2.70)	0.084
Type of house				
Independent/Flat	Ref.		Ref.	
Tent/Hut	0.56 (0.43–0.74)	<0.001	0.76 (0.55–1.07)	0.114
Family owned television				
No	Ref.		Ref.	
Yes	1.42 (1.07–1.88)	0.014	1.04 (0.75–1.43)	0.812
Type of family				
Host community	Ref.		Ref.	
Internally displaced	0.52 (0.39–0.70)	<0.001	0.73 (0.52–1.04)	0.079

COR, crude odds ratio; CI, confidant intervals; AOR, adjusted odds ratio; Ref, reference group.

Table 5 presents the association between mothers’ good knowledge of the routes of diarrhea transmission and prevention and the management of acute diarrhea among children. In the bivariate analysis, mothers with better knowledge were nearly twice as likely to provide ORS compared with other traditional methods (COR = 1.73; 95% CI: 1.19–2.50). There was no association between mothers’ knowledge and visiting health facilities (COR = 0.85; 95% CI: 0.60–1.20). This relationship remained unchanged after adjusting for mothers’ age, education, occupation, and type of family (IDPs or host community). Mothers with good knowledge were still nearly twice as likely to provide ORS (AOR = 1.69; 95% CI: 1.15–2.49).

**Table 5:** Association between mothers’ knowledge and the management of acute diarrhea in children.

Management of diarrhea in children	COR (95% CI)	*P*. value	AOR (95% CI)	*P*. value
Methods				
Traditional methods	Ref		Ref	
Providing oral rehydration solution	1.73 (1.19–2.50)	0.004	1.69 (1.15–2.49)	0.007
Visiting health facilities	0.85 (0.60–1.20)	0.356	0.94 (0.65–1.34)	0.722

COR, crude odds ratio; CI, confidant intervals; AOR, adjusted odds ratio; Ref, reference group.

## Discussion

The 36.4% prevalence of acute diarrhea among vulnerable children from internally displaced persons and host communities in this study aligns with the global prevalence of diarrhea (29%) reported among internally displaced and refugee camp populations (29). However, it is lower than the rates reported for internally displaced children in Somalia (51%) (30) and higher than 26.6% from Ethiopia (31). Furthermore, the prevalence of acute watery diarrhea among children in this study differed by current place of residence. This difference may be explained by greater exposure to risk factors, such as inadequate water, sanitation, and hygiene conditions, among the children in our study compared with children in the general populations examined in those studies, as well as differences among children living in different areas within this study.

Around 40.0% of displaced and host mothers self-reported good knowledge about diarrhea in children. This rate is close to 37.5% of mothers in indigenous and resettlement communities in Ethiopia who have good knowledge of the causes, transmission, and prevention of diarrhea in children (32). On the other hand, this rate is lower than those reported in a study carried out among refugees and host communities in Ethiopia (33) and from a finding of studies conducted among general population in Saudi Arabia (34), Ethiopia (19), and Nigeria (20). The low proportion of mothers with good knowledge observed in this study may be attributed to several factors. First, high levels of illiteracy in these communities can limit mothers’ ability to understand and retain health information. Second, limited access to the internet and social media reduces exposure to health education messages and health promotion campaigns. Finally, inadequate access to health facilities may further restrict mothers’ opportunities to obtain health-related information from healthcare providers.

In line with a Congo study (35), this study found that mothers aged 40 years or older had better knowledge about childhood diarrhea. This may be because older mothers have learned more over time and gained experience, making them more knowledgeable about the causes, transmission, and prevention of diarrhea. Further, this study agrees with studies from Congo (35), Pakistan (36), and Tanzania (37), which found a link between mother’s education level and their knowledge about childhood diarrhea. Educated mothers may have better access to health messages from schools, media, and health care services, which can increase their awareness. While mothers who work outside the home were significantly more likely to have good knowledge about diarrhea in bivariate analysis, this association disappeared in multivariable analysis. This may be due to the confounding effect of age, since working mothers may be more likely to be older and older women generally have greater experience and knowledge. Once age and other factors were controlled for, the independent effect of employment status was no longer significant. This finding is inconsistent with study from Ethiopia that found association between maternal occupation and their knowledge about diarrhea (38).

Regarding the management of childhood diarrhea, the proportion of mothers who use ORS (15.9%) in this study is lower than 64.6% in Nigeria (20), 76.5% in Nepal (39), and 85.3% in Bangladesh (40). ORS has been shown to reduce symptom severity and duration and to lower the risk of recurrence in the short term (41, 42). ORS could prevent an estimated 525,000 deaths among under-five children from diarrhea each year (42). Moreover, the proportion of mothers who seek healthcare for children with diarrhea in this study is lower than that reported Ethiopia (43), sub-Saharan Africa (44), and LMICs (45) (73.8%, 58.7%, and 52.5%, respectively). Delays in accessing healthcare can significantly affect children’s health, reduce the effectiveness of treatments (43, 46, 47), and increase the risk of fatalities among children (48). In line with other studies (39, 49–51), the findings of this study revealed that mothers with good knowledge of diarrhea were almost twice as likely to provide ORS. This suggests that maternal knowledge plays a significant role in caregivers’ decision-making and supports the adoption of recommended home-based treatment practices. Conversely, the lower likelihood of ORS provision among mothers with poor knowledge may be attributed to limited awareness of ORS and to an underestimation of the seriousness of diarrhea in children. Mothers’ knowledge, on the other hand, did not show an association with visiting health facilities, which may be due to the fact that health facilities are located far from these communities (22).

The findings of this study have important implications for the prevention and control of childhood diarrhea. The high proportion of mothers with poor knowledge about the mode of transmission and prevention of diarrhea highlights a critical gap in preventive practices that may contribute to ongoing transmission within communities. In addition, the low level of healthcare-seeking and inadequate use of recommended diarrhea treatments such as ORS suggest that many children may not receive timely and appropriate care, increasing the risk of dehydration and complications. Therefore, there is a need to strengthen community-based health education interventions that specifically target caregivers’ understanding of how diarrhea spreads and the key behaviors that can prevent it, including safe water, proper sanitation, and appropriate hygiene. Moreover, since geographical remoteness and long distances to health facilities may delay treatment, program efforts should focus on improving access through community health workers, outreach services, and strategies that enhance referral and follow-up for under-five children.

One limitation of this study is its cross-sectional design, which allows the identification of associations but does not establish causality between the study factors and mother’s knowledge about childhood diarrhea. In addition, the study used self-reported data, which may be influenced by recall bias or social desirability bias. One key strength of this study is that it was community-based and conducted in war-affected areas across four governorates. The study also included internally displaced persons and host community members living in remote areas. This broad and diverse coverage improves the relevance of the findings to different populations affected by displacement and conflict.

## Conclusion

In conclusion, 36.4% of children under the age of five had acute watery diarrhea. The study highlights critical gaps in mothers’ knowledge and health-seeking behaviors regarding childhood diarrhea in vulnerable population. Mother’s age and education play a role in increasing mothers’ knowledge. Given these findings, it is essential to implement targeted educational interventions that not only increase mother’s awareness of the route of transmission and the prevention of diarrhea, but also encourage the adoption of effective treatment methods. Further studies exploring the factors influencing mothers’ health-seeking behaviors for children with acute diarrhea are recommended.

## Data Availability

The original contributions presented in the study are included in the article/Supplementary Material, further inquiries can be directed to the corresponding author.
